# Saliva substitute mouthwash in nasopharyngeal cancer survivors with xerostomia: a randomized controlled trial

**DOI:** 10.1007/s00784-020-03634-5

**Published:** 2020-11-11

**Authors:** Dayaabaran Marimuthu, Kong Min Han, Mohd Shawal Firdaus Mohamad, Mawaddah Azman

**Affiliations:** 1 Department of Otorhinolaryngology and Head and Neck Surgery, Level 9, Clinical Block, UKM Medical Centre, National University of Malaysia Kuala Lumpur Campus, Jalan Yaacob Latiff, Bandar Tun Razak, 56000 Kuala Lumpur, Malaysia; 2 Centre of Studies for Oral and Maxillofacial Surgery, Faculty of Dentistry, UiTM Sungai Buloh Campus, Jalan Hospital, 47000 Sugai Buloh, Selangor Malaysia

**Keywords:** Xerostomia, Mouth dryness, Hyposalivation, Saliva artificial, Saliva substitute, Mouthwashes, Radiation-induced toxicity, Nasopharyngeal cancer

## Abstract

**Objectives:**

Xerostomia is a prevalent sequelae among nasopharyngeal cancer (NPC) survivors; yet, effective treatment protocols have been elusive. This study was a prospective randomized clinical trial to compare the effects of saliva substitute mouthwash in nasopharyngeal cancer survivors with xerostomia, between two treatment arms, conducted in a tertiary center.

**Materials and methods:**

This study measured the effects within 4 weeks in relation to summated xerostomia inventory (SXI) and unstimulated whole saliva (UWS). Patients randomized into the interventional arm were prescribed an immunologically active saliva substitute (IASS), while patients in the control arm were prescribed a non-immunologically active mouthwash as placebo.

**Results:**

The study population consisted of 94 patients. There was a significant difference in SXI difference (*p* < 0.0001) and UWS difference (*p* < 0.0001) between control and interventional arms. No harmful side effects associated with the use of either mouthwash encountered throughout the study duration.

**Conclusion:**

IASS mouthwash significantly reduces subjective xerostomia scores measured using SXI and improves objective measurement of salivary flow using UWS among nasopharyngeal cancer survivors with xerostomia.

**Clinical relevance:**

IASS is significantly more effective in improving subjective and objective xerostomia measurements compared to non-immunologically active mouthwash. Additionally, this treatment is very safe, with superior side effect profiles.

**Trial registration:**

ClinicalTrials.gov Identifier: NCT04491435

## Introduction

Nasopharyngeal carcinoma (NPC) is the highest reported otorhinolaryngological malignancy in Malaysia affecting predominantly male adults between 40 and 60 years old [1, 2]. Radiation therapy (RT) has been coined as the mainstay treatment owing to its’ radiosensitive properties [1, 3]. Radiation-induced DNA damage impairs proper cell division, resulting in cell death or senescence of cells that attempt to divide, particularly useful in killing malignant cells. However, radiation doses to the salivary glands cause loss of saliva producing acinar cells which ultimately hampers production of saliva in NPC patients post radiation [4]. This leads to progressive loss of salivary gland function causing xerostomia symptoms [5].

Xerostomia, or dry mouth caused by reduced or absent saliva flow, is a subjective symptom that can lead to impaired chewing, swallowing, altered sense of taste, and speech. This eventually affects their nutritional status and quality of life. The reported prevalence of xerostomia in NPC survivors ranged from 80 to 100% [6–10]. This high prevalence has stemmed a lot of interest in prevention and treatment of this important sequelae. Palliative treatments such as mucosal lubricants, saliva substitute, saliva stimulants, and systemic or local sialogogues have been proposed [11–13].

Saliva substitute can be classified based on its active ingredients: carboxymethylcellulose, glycerol, immunologically active saliva substitute, xanthan, and herbal preparations [14]. They are commercially available in the forms of gels, toothpaste, sprays, or mouthwash [15]. Given that a wide variety of saliva substitute preparations are available, either as prescriptions or over the counter medications, good quality randomized trials are necessary to investigate its effectiveness in specific patient populations. A recent review in 2019 on saliva substitute included 21 studies conducted on various patient populations, a mere 4 trials in radiation-induced xerostomia and only 2 non-randomized trials on immunologically active saliva substitute [14]. The paucity of data on clinical effectiveness of saliva substitute in patients with radiation-induced xerostomia is rather compelling and warrants further research.

Oral7® mouthwash (Oral7 International, UK) is an immunologically active saliva substitute (IASS) formulated with natural enzymes such as lactoperoxidase, lysozyme, glucose oxidase, and lactoferrin, similar to naturally occuring saliva. The biophysical properties of the mouthwash can potentially provide relief to xerostomia symptoms in patients post radiotherapy translating to a better quality of life. Hence, the purpose of the present study is to evaluate the effects of IASS in treating xerostomia among NPC patients post radiotherapy. The primary end-point of the study was to compare the subjective xerostomia symptoms, measured using a validated inventory taken 4 weeks following intervention and at baseline between patients who did not receive and patients who received Oral7® mouthwash. The secondary end-point was to compare unstimulated whole salivary flow measurements using sialometry technique between patients who did not receive and patients who received Oral7® mouthwash after a treatment duration of 4 weeks.

## Materials and methods

**Fig. 1:**
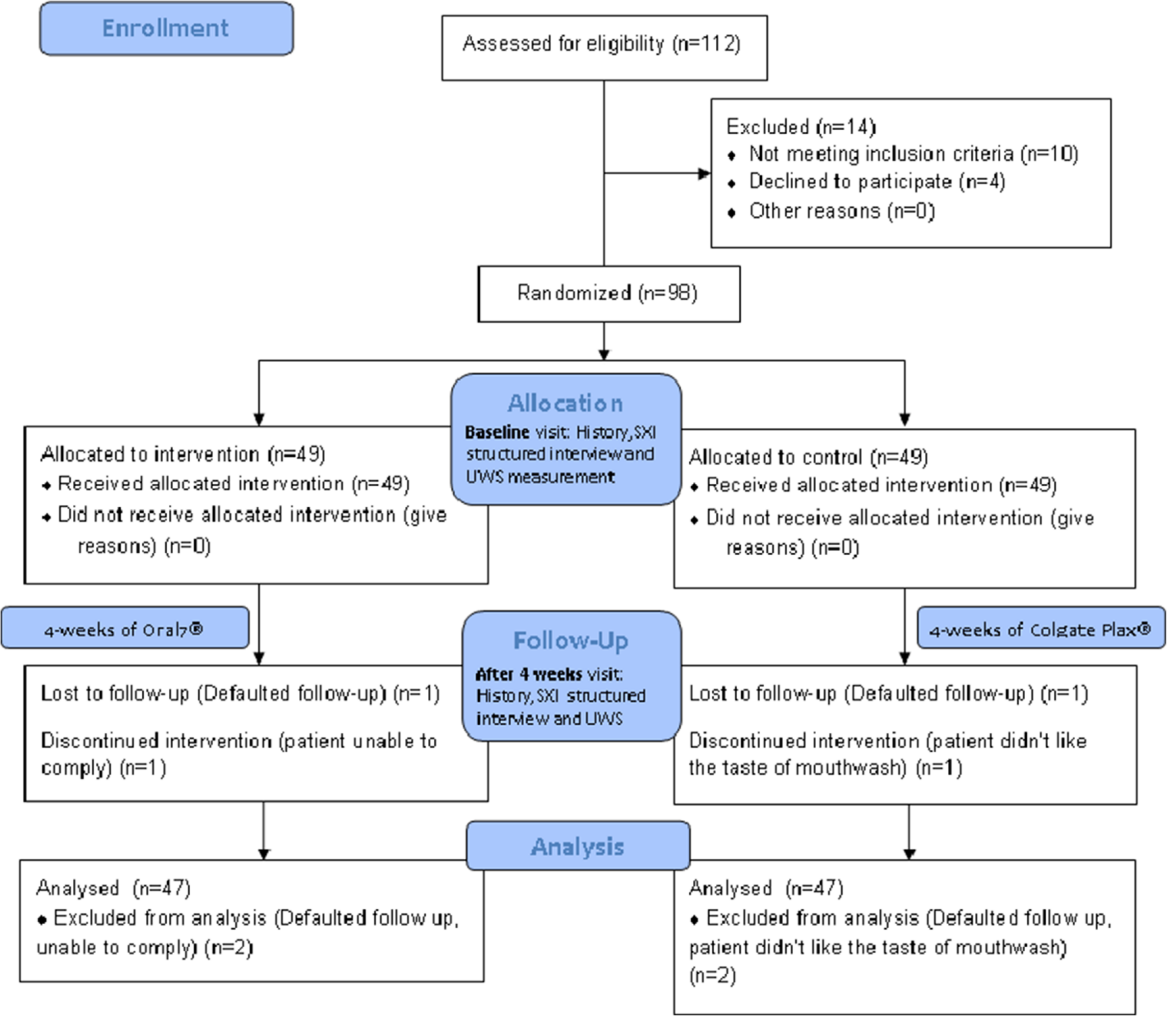
Study flow

The efficacy of the saliva substitute Oral7® mouthwash was evaluated in a prospective, double-blind, randomized, placebo-controlled study that was conducted in the Department of Otorhinolaryngology, Universiti Kebangsaan Malaysia Medical Centre, a tertiary referral center between June 1, 2018 (date of first patient recuitment) and January 2, 2020 (date of last patient follow-up) for a duration of 19 months. The study received partial funding from the Fundamental Grant of National University of Malaysia and the local distributor provided the Oral7® mouthwash used in this study. This study was approved by the Institutional Review Board of National University of Malaysia, with the project code FF-2018-133, along with its financial support on May 5, 2018. The conduct of the study followed the Declaration of Helsinki 1964 and the authors have no financial or affiliations with the distributors of the product to disclose. This study was designed, analyzed, and interpreted according to the CONSORT protocol (Fig. 1) [16].

All consecutive patients diagnosed with nasopharyngeal carcinoma during the study period who underwent either radiotherapy or concurrent chemo-radiotherapy were screened for the study. Patients who satisfied the inclusion and exclusion criterias (Table 1) were recruited. Written informed consent was obtained from the patients for publication of this manuscript. A copy of all the written consents is available for review by the Editors-in-Chief of this journal.

**Table 1:** Inclusion and exclusion criteria

Inclusion criteria	
1. Patients who have completed radiation therapy or concurrent chemotherapy and radiation therapy for nasopharyngeal carcinoma (clinical staging of tumor based on AJCC staging (8^th^ edition) *T*_1–4_, *N*_0–3_, *M*_0_).	
2. Patients age 20 years old to 85 years old.	
3. Two months has elapsed since last dose of chemotherapy or radiotherapy.	
4. Karnofsky performance score more than 70%.	
5. Patients complaining of xerostomia.	
Exclusion criteria	
1. Those contraindicated to using mouthwash (established allergy to lactoperoxidase, lysozyme, glucose oxidase lactoferrin, cetylpyridinium chloride, and xylitol).	
2. Patients with residual or recurrent disease.	
3. Patients who received intensity-modulated radiation therapy.	
4. Patients with ongoing oral mucositis (WHO Oral Mucositis grading I to IV).	
5. Patients with facial, glossopharyngeal. vagus and hypoglossal nerve palsy/ paresis.	
6. Patients who had any form of concurrent treatment protocols (hormonal, alternative, antiviral, sialogogues, or photodynamic therapy) during the study duration.	
7. Patients with autoimmune diseases such as systemic lupus erythematosus, rheumatoid arthritis, and Sjớgren syndrome.	

**Fig. 2:**
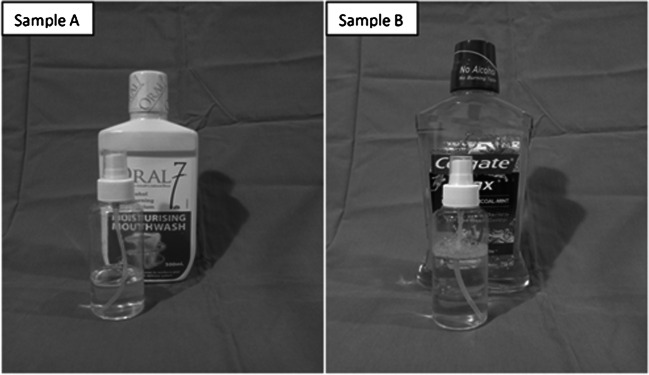
Sample A and sample B mouthwashes dispensed to the study population

A randomization list was generated from the statistics website http://www.graphpad.com/quickcalcs/index.cfm where consecutive patients enrolled into the study were randomized to intervention and control arms with an allocation ratio of 1:1. The randomization list was generated by the lead researcher, who was not involved in enrolling, assigning the patients, and collection of data. The researcher assigning the participants to intervention arms did not know the allocation sequence until the moment of assignment. This way, the researcher was prevented from (consciously or unconsciously) influencing which participants who were assigned to a given intervention arm. Patients were dispensed sample A (saliva substitute containing Oral7® mouthwash) or sample B (placebo containing Colgate Plax®), in similar non-pressurized pumps (Fig. 2) according to the randomization list.

Oral7® mouthwash (Oral7 International, UK) is an immunologically active saliva substitute (IASS) formulated with natural enzymes such as lactoperoxidase, lysozyme, glucose oxidase, and lactoferrin, similar to naturally occuring saliva. The mouthwash in liquid form was repackaged into specific non-pressurized pumps measuring 90 g each and was labeled sample A. Sample A was dispensed to patients recruited to the interventional arm.

Colgate Plax® (Colgate-Palmolive, Malaysia) is a mouthwash with biocidal properties containing cetylpyridinium chloride as the main active ingredient. This mouthwash was with no alcohol, no mucin, and no natural enzymes, making it a suitable placebo for this study. Additionally, the mouthwash contains xylitol, which provides some moisturizing effects, suitable for use in NPC survivors with xerostomia. The mouthwash in liquid form was repackaged into specific non-pressurized pumps measuring 90 g each and was labeled sample B. Sample B was dispensed to patients recruited to the control arm. Samples A and B possessed similar physical characteristics, such that the patients were blinded to the intervention that they received (Fig. 2).

The samples were dispensed by a dedicated nurse who was not part of the study. Both the researcher and patient are blinded to the type of intervention they received. The randomization list was not known to the researcher measuring the study outcome measures until data was analyzed. Patients were instructed to use the mouthwash by spraying 2 puffs of either samples A or B, three times a day. They were instructed to discard the samples if it had discolored or contained particulate material. Side effects experienced were collected through patient interviews either during weekly follow-up phone calls or clinic visits. The patients were not allowed to use other mouthwashes and hormonal or nutritional supplements throughout the study period. At the first visit, patients were given 2 weeks’ supply of mouthwash in a designated box. Patients were instructed to bring the designated box with the remaining samples at the subsequent 2-week visit. They were told that more than enough samples were dispensed in order to encourage them to return unused samples. The number of samples left in the box and the weight of samples in each non-pressurized pump were measured at each visit and freshly measured samples were replenished at the end of 2 weeks. To further ensure patient compliance, weekly phone calls were made to all patients.

Demographic data including age, gender, education level, occupation, date of treatment completion, and relevant medical history such as type of treatment, Karnofsky performance score, histopathological type, and underlying medical illness were recorded. At the first visit, all patients underwent a structured interview by a dedicated researcher to obtain a baseline score of the summated xerostomia inventory (SXI). During the same visit, unstimulated whole saliva (UWS) flow was assessed by the same dedicated researcher using sialometry technique. All patients were instructed to use the saliva substitute (sample A) or placebo (sample B) for 4 weeks and the SXI and UWS were reassessed 4 weeks later. The flow of the study is summarized in Fig. 1. Follow-up phone calls were performed on a weekly basis to ensure compliance and record potential side effects that the patients may have experienced when using either samples A or B. Weight of returned empty pumps were documented to record compliance in recruited patients.

### Sample size calculation

The sample size calculation for this interventional study was performed using Power and Sample Size (PS3) software (2009) by Dupont and Plummer from Vanderbilt University. The method used was the formula for prospective studies with dichotomous outcome and analyzed by *t* tests. Ninety-four patients with 47 patients from the interventional population were needed to make the study statistically significant with a 95% confidence interval to detect significant differences between the control and interventional groups.

### Data collection and analysis

Demographic data, including patients’ age, sex, and race, were obtained from patient interviews. Cancer type, staging, and oncologic treatment performed were obtained from patients’ records, radiological reports, or the final pathological report from the Health Information System. Patients’ age was grouped according to Erik-Eriksson’s psychological stages of life [17]. Tumors were staged based on the American Joint Committee for Cancer (8th Edition) [18].

Subjective xerostomia score was measured using summated xerostomia inventory (SXI). The Dutch version of the SXI was used as a primary outcome measure. The SXI contained 5 questions and was chosen as a more suitable questionnaire compared to the longer xerostomia inventory (XI) with 11 questions. Thomson et al. looked at different studies that compared the validity of SXI to XI which showed properties of the scale were not compromised by reducing the number of response options available to respondents. The authors suggested that the shorter measure has considerably better face validity than the original, because the items were more salient [19]. The SXI score was assessed in a structured interview by a dedicated researcher, each question provided in Table 2. For each question, patients were instructed to choose one of three responses to describe their subjective response past one week, either “Never” (score 1), “Occasionally” (score 2), and “Often” (score 5). The total score was calculated for both baseline and 4 weeks following intervention visits. Total scores ranged from 5 (no xerostomia) to 25 (worst possible xerostomia). The difference between the total SXI score taken at baseline and after 4 weeks of intervention was calculated and used as an outcome measure.

**Table 2:** Summated xerostomia inventory (SXI) questions

Item	Question	Score (Never (1), Occasionally (2), Often (5))
1	My mouth feels dry when eating a meal	
2	My mouth feels dry	
3	I have difficulty in eating dry foods	
4	I have difficulty swallowing certain foods	
5	My lips feel dry	
	Total score	

Sialometry was performed by a trained dedicated researcher, at the beginning of the study and 4 weeks after intervention. Patient was seen in the clinic and reminded not to take any food, water, or smoke 1 h prior to saliva collection. After mouth-rinsing with 10 mls sterile water at room temperature, unstimulated whole saliva (UWS) was collected by putting 2 pieces of standardized dental roll underneath the patient’s tongue. The dental rolls were pre-weighed prior to placing it in the patient's mouth. After 5 min, the dental rolls were removed and weighed; patients were also told to spit any amount of saliva in their mouth to a pre-weighed container. Patients were supervised by the researcher and reminded not to swallow any saliva during the collection period. The weight of the UWS was recorded in milligram (mg). The salivary flow rate was expressed as ml/min, according to the method described by Navazesh and Christensen [20]. Measurements were made immediately after saliva collection. The difference between the UWS measurement taken at baseline and after 4 weeks of intervention was calculated and used as an outcome measure.

Statistical analysis was performed using SPSS statistical analysis software, version 23.0 (SPSS Inc., Chicago, IL). Test for normality was performed using the Shapiro-Wilk test. Since all parameters did not show a normal distribution, non-parametric statistics were applied to group demographic analysis, SXI and UWS difference. Descriptive analysis was carried out and variables were described as median (IQR). Comparison of primary and secondary end points (SXI and UWS difference) was performed using Mann-Whitney *U* test. Multiple group comparisons were performed using Fisher’s exact and Kruskall-Wallis test. *p* < 0.05 at 95% CI was regarded as statistically significant.

## Results

### Demographic characteristics

Ninety-eight patients who fulfilled the inclusion and exclusion criteria were enrolled in this study. Four patients were excluded, making a final study population of 94 patients. Two patients (one from control and interventional arm each) had to be excluded from the study as they could not attend the follow-up visit despite multiple reminders, and the other two (one from control and interventional arm each) requested to be withdrawn from the study 2 weeks after enrolment. One patient from the interventional arm was unable to comply with the instructions given while the other patient from the control arm did not like the taste of the placebo mouthwash. Data from a total of 47 patients in the interventional and control arm, each were analyzed. The median age was 58 years (range, 27–81 years). Among 94 patients, 63 were men (67.0%) and 31 were women (33.0%). The patients were from different ethnicities in which 26 were Chinese (59.1%) and 14 were Malay (31.8%). Shapiro-Wilk test showed that the data was not normally distributed; hence, non-parametric tests were used for subsequent data analyses.

Table 3 summarizes demographic characteristics, Karnofsky performance score, highest level of education, heterogeneous radiological staging, treatment received, and years after completion of treatment in our study population. Fisher’s exact test comparing specific characteristics like gender, ethnicity, age group, Karnofsky performance score, highest level of education, and staging and type of treatment received did not reveal any significant differences between the control and interventional arms (*p* > 0.05). Analysis using Kruskal-Wallis test was performed for years after completion of treatment. No significant differences were seen comparing these potential confounders between the control and interventional arms. The study population was homogeneously distributed across the 2 arms of the study with no significant differences found between specific demographic characteristics (Table 3).

**Table 3:** Demographic characteristics of study population

Demographic characteristic	Control *n* = 47 no. of patients (%)	Interventional *n* = 47 no. of patients (%)	Overall *n* = 94 no. of patients (%)	*p* value
Gender				
Male	34 (72.3%)	29 (61.7%)	63 (67.0%)	0.739*
Female	13 (27.7%)	18 (38.3%)	31 (33.0%)	
Ethnic				
Malay	10 (21.3%)	15 (31.9%)	25 (26.6%)	1.000*
Chinese	37 (78.7%)	32 (68.1%)	69 (73.4%)	
Age group				
Young and Middle Adult	33 (70.2%)	38 (80.8%)	71 (75.6%)	0.337*
Elderly	14 (29.8%)	9 (19.2%)	23 (24.4%)	
Karnofsky performance score				
80	15 (31.9%)	4 (8.5%)	19 (20.2%)	0.931*
90	29 (61.7%)	37 (78.7%)	66 (70.2%)	
100	3 (6.4%)	6 (12.8%)	9 (9.6%)	
Highest education				
Primary	9 (19.1%)	9 (19.1%)	17 (38.6%)	0.838*
Secondary	26 (55.3%)	28 (59.6%)	4 (9.1%)	
Tertiary	12 (25.5%)	10 (21.3%)	2 (4.6%)	
Stage				
I	8 (17.0%)	4 (8.5%)	11 (25.0%)	0.562*
II	22 (46.8%)	25 (53.2%)	1 (2.3%)	
III	15 (31.9%)	14 (29.8%)	2 (4.6%)	
IV	2 (4.3%)	4 (8.5%)	2 (4.6%)	
Treatment received				
Radiotherapy alone	3 (6.4%)	5 (10.6%)	8 (8.5%)	1.000*
Concurrent chemoradiation	44 (93.6%)	42 (89.4%)	86 (91.5%)	
Years after completed treatment				
0–5	26 (55.3%)	26 (55.3%)	52 (55.3%)	0.876^✝^
5–10	15 (31.9%)	8 (17.0%)	23 (24.5%)	
> 10	6 (12.8%)	13 (27.7%)	19 (20.2%)	

*Fisher’s exact test

^✝^Kruskall-Wallis test

### SXI score

**Fig. 3:**
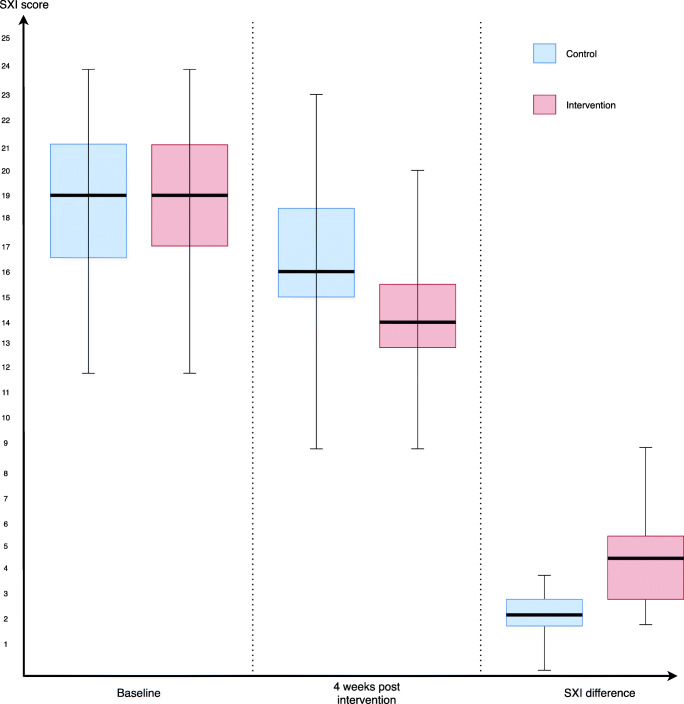
Box plot showing SXI comparison between treatment arms

Measurements taken at baseline showed that, majority of patients scored “Often” for the question “I have difficulty in eating dry foods” in both arms. Meanwhile, 98% of the patient scored “Never” for the question “My lips feel dry.” Median of baseline SXI score for the control arm was 19 (IQR = 2) similar to the interventional arm at 19 (IQR = 4) (Fig. 3). No significant difference in baseline SXI score was found between the 2 arms (*p* = 0.658). This meant the severity of xerostomia between both groups were identical at baseline. At 4 weeks following intervention, the median of total SXI score for the control arm was 16 (IQR = 3.5), meanwhile for the interventional arm was 14 (IQR = 2.5). Significant difference was seen in between SXI score at 4 weeks between the 2 arms (*p* = 0.002). SXI difference between 4 weeks and baseline was calculated for all patients (SXI difference = total SXI score at 4 weeks – total SXI score at baseline). The median difference of SXI was 2 (IQR = 1) for control arm while for interventional arm, the median difference was 4 (IQR = 2.5) (Fig. 3). All 47 patients in the interventional arm had improved xerostomia symptoms at 4 weeks in contrast to 2 patients in the control arm who had no improvement in xerostomia symptoms. *p* value obtained using Mann-Whitney *U* test showed a significant difference between the 2 arms (*p* < 0.0001). Patients in the interventional arm showed substantial improvement in subjective xerostomia symptoms compared to those in the control arm.

### Sialometry value

**Fig. 4:**
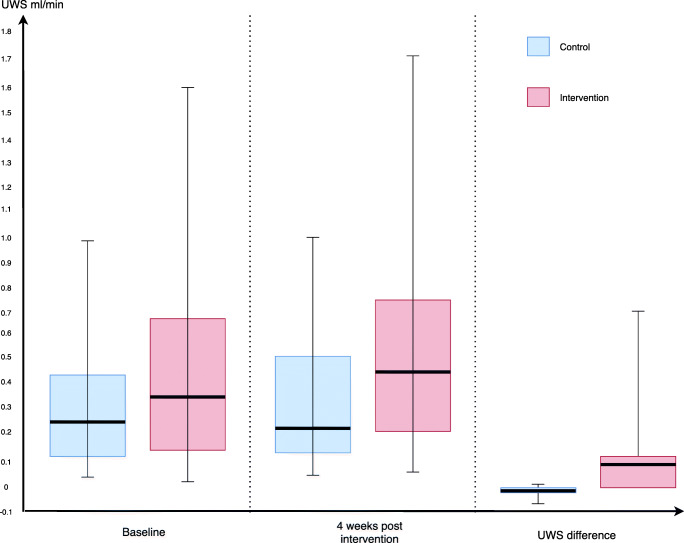
Box plot showing UWS comparison between treatment arms

Median of baseline UWS flow for the control arm was 0.249 (IQR = 0.389) ml/min and 0.348 (IQR = 0.535) ml/min for interventional arm (Fig. 4). No significant difference in baseline UWS flow was found between the 2 arms (*p* = 0.157). At 4 weeks following intervention, median UWS flow for the control arm was 0.240 (IQR = 0.389), meanwhile for the interventional arm was 0.453 (IQR = 0.535). Significant difference was seen in between UWS flow at 4 weeks between the 2 arms (*p* = 0.006). UWS difference between 4 weeks and baseline was calculated for all patients (UWS difference = UWS flow at 4 weeks – UWS flow at baseline). The median difference of UWS was 0.002 (IQR = 0.005) ml/min for control arm while for interventional arm the median difference was 0.046 (IQR = 0.101) ml/min (Fig. 4). All 47 patients in the interventional arm had improved unstimulated whole salivary flow at 4 weeks in contrast to 11 patients in the control arm who suffered worsening of unstimulated whole salivary flow. *p* value obtained using Mann-Whitney *U* test showed a significant difference between the 2 arms (*p* < 0.0001). Patients in the interventional arm showed substantial improvement in unstimulated whole salivary flow compared to those in the control arm.

### Harms or unintended effects

Throughout the study duration, no unintended harms were encountered following the use of either samples A or B among our study population. All patients were compliant to the use of the study samples. There were no reported allergies with the use of the samples throughout the 4 weeks’. However, one patient randomized to the control arm decided to drop out from the study after using sample B for 2 weeks. The reason for withdrawal was unpleasant taste profile for sample B. This unintended effect was short lived and stopped immediately following discontinuation of treatment. No hospitalization was necessary and no other side effects were documented.

## Discussion

Xerostomia is an important and prevalent complication seen among NPC survivors with a reported prevalence ranging 80–100% [6–9]. It is a subjective symptom caused by reduced or absent saliva. Xerostomia leads to impaired chewing, swallowing, altered sense of taste, and speech in NPC survivors. This would eventually affect their nutritional status and ultimately the quality of life. Various treatment strategies for the management of xerostomia have been proposed in the past to reduce patients’ symptoms and/or increase salivary flow. Medications such as mucosal lubricants, saliva substitutes, saliva stimulants, and systemic/local sialogogues have been proposed in recent literature [11–13].

Our study compared the efficacy of the saliva substitute Oral7 ® mouthwash against placebo in treating xerostomia post radiotherapy in NPC patients. The results support our hypothesis that immunologically active saliva substitute (IASS) can reduce xerostomia symptoms in NPC patients post radiotherapy. Both the subjective and objective outcome measures in the intervention arm showed a statistically significant difference compared to the control arm. SXI scores in patients using Oral7® showed a significant reduction in all patients with a median of 4 (IQR = 2.5). Meanwhile, for patients in the control group, there was a reduction of scores in certain patients with a median of 2 (IQR = 1). Patients in the Oral7® arm showed an improvement of UWS with a median difference of 0.046 (IQR = 0.101) ml/min compared to those in the control arm where the median UWS difference was 0.002 (IQR = 0.005) ml/min. This shows Oral7® not only improved patients’ xerostomia symptoms but also increased their salivary flow.

The present study was a randomized, placebo-controlled trial studying the efficacy of a saliva substitute mouthwash conducted in a tertiary center. Potential confounders like age group, years after completion of treatment, Karnofsky performance score, tumor stage, and type of treatment received were compared using Fisher’s exact test and Kruskal-Wallis test between the two treatment arms. All mentioned confounders were homogenously distributed between the interventional and control arms.

We compared Oral7® mouthwash used by patients allocated to the interventional arm, and Colgate Plax® used by patients allocated to the placebo arm. Colgate Plax® does not contain natural enzymes, but it possesses biocidal properties with some moisturizing effect, advantageous to our study population. Although physical properties such as color and taste profiles are not exact matches to Oral7® mouthwash, further blinding was provided by repackaging the two solutions into similar non-pressurized pumps, making it hard for the patients to identify these different mouthwashes. Colgate Plax® is a suitable placebo to be used considering that it is not a recognized treatment for xerostomia. The present study is the first to use Colgate Plax® as a placebo. Other published studies have used sodium fluoride solution and salt-soda mouthwash as placebo [21, 22].

A pilot study of 30 patients by Jellema et al. studied the efficacy of xialine, a type of saliva substitute in a placebo-controlled randomized cross-over study using EORTC QLQ-C30 and the QLQ-H&N35 questionnaires. In this study, a small but not statistically significant improvement was demonstrated in the global quality of life (QoL) scores after 1 week of treatment with either xialine or placebo [23]. Although the cross-over trial design was better compared to the present study, the duration of treatment was perceived to be too short, and desired outcomes could not be observed. The present study recruited more patients and increased the treatment duration (4 weeks) to rigorously assess the effects of the saliva substitute Oral7® mouthwash. Future cross-over trials utilizing similar or longer treatment durations would consolidate the findings of our study, but could suffer from higher drop out.

The summated xerostomia inventory was used in this present study as a primary outcome measure. This inventory was adapted from the SXI-Dutch version which has been shown to be a valid tool for measuring xerostomia symptoms in a clinical research [19]. Following validation of the summated English version, it has been used widely in many clinical and epidemiological studies on xerostomia [24, 25]. In the present study, this inventory was administered using a structured interview by a dedicated researcher, which exposes the study to the risk of evaluation apprehension. To utilize a patient-administered questionnaire, the multi ethnic study population called for a validated inventory in Malay and Mandarin languages which was unavailable at the time of study design [26, 27]. Validation of the SXI-Dutch version in Malay and Mandarin languages is a potential avenue for further research as it will facilitate the delivery of this inventory as an outcome measure in future trials. In contrast to QLQ-H&N35 questionnaires, the summated xerostomia inventory has more focused questions addressing xerostomia symptoms, making it less time consuming for the patients. The absence of global QoL scores for further analysis did not affect the study results as it is well documented that xerostomia adversely affects QoL outcomes in NPC survivors [10].

Various methods for objective measurement of salivary flow, an important outcome measure in xerostomia research have been reported and tested for validity and reproducibility [20, 28–30]. The present study measured unstimulated whole saliva (UWS) using a method described by Navazesh and Christensen. Measurement of salivary flow from individual salivary glands, although more accurate is time consuming, laborious, and require custom-made collection devices [31]. Additionally, cannulating the salivary ducts can potentially introduce ascending infection to our already vulnerable study population. A later systematic review by Löfgren et al*.* evaluating methods to measure salivary flow using Quality Assessment of Diagnostic Accuracy Studies (QUADAS) was unable to conclude a gold standard method of evaluating salivary flow [32]. Although the original study by Navazesh and Christensen reported only acceptable reliability (test-retest correlation coefficient = 0.68) using the UWS collection method, the study population consisted of only 17 healthy volunteers. During conception of this current study, UWS was chosen as an outcome measure because it is non-invasive, less time consuming and widely used in other published literature [22, 33–36].

Normal salivary flow is an important prerequisite for homeostasis of the oral environment. With the introduction of radiation to treat malignant pathologies of the upper aerodigestive tract, the patient will suffer loss of 50–70% of saliva function if 10–15 Gy of radiation is applied or even worse it is undetectable when higher doses of radiation (40–42 Gy) is given to the patient [37]. There have been numerous published studies on the efficacy of saliva substitute in treating xerostomia [14, 38–44]. Studies that looked into the efficacy of Oral7® mouthwash in treating xerostomia in post radiotherapy patients is, however scarce [21]. Bachok et al. in their preliminary randomized trial of 30 patients undergoing radiotherapy for head and neck cancer comparing Oral7® mouthwash and salt-soda mouthwash reported significant improvement in QoL scores at 3 months post-treatment. Although the study included sialometry measurements as secondary end points, this outcome measure was not reported. The findings of our study showed that at 4 weeks post intervention, patients treated with Oral7® mouthwash had a median UWS of 0.453 (IQR = 0.535) ml/min, which was significantly higher compared to those treated with placebo. As many as 23 (49%) patients in the interventional arm achieved a normal UWS more than 0.5 ml/min at 4 weeks post intervention. With these findings, we believe that Oral7® mouthwash is beneficial to maintain the unstimulated salivary flow to a near-normal state.

Findings of this study are consistent with most previous studies on saliva substitute used in various study populations with xerostomia [39–43]. The saliva substitute tested comprised agents such as immunologically active natural enzymes (current study), carboxylmethy cellulose, mucin, and glycerin [14, 40–43]. To date, excluding the earlier trial by Bachok et al., there are two other studies investigating the efficacy of immunologically active natural enzymes. BioXtra gel containing lysozyme was studied in a non-randomized trial among 58 patients with head and neck cancer where improved xerostomia symptom score after 2 weeks use was reported [45]. Another randomized study in 134 diabetic patients treated with 6 months of immunologically active natural enzymes showed significant improvement in dental plaque index and yeast culture, which may shed light to additional benefit of long term treatment with this type of saliva substitute [44]. Findings of these studies corroborated that immunologically active natural enzymes is a safe and efficacious salivary substitute to use in various study population with xerostomia [14, 21, 44, 45]. At the time of writing, the present study was the first to document improved UWS flow with 4 weeks’ use of immunologically active natural enzymes.

Our results support the clinical use of Oral7® mouthwash in alleviating xerostomia symptoms among NPC survivors. Hence, we would like to recommend continued use of the saliva substitute to obtain desired clinical outcomes in these patients. The findings of this study can be extrapolated for clinical application in other diseases presenting with xerostomia such as Sjögren syndrome or drug induced xerostomia. However, we did not evaluate whether sustained effects can be seen following discontinuation of use; hence, this cannot be considered a permanent treatment. Albeit this treatment avenue does not require any surgical intervention to alter the glands such as glandopexy or tissue regenerative methods, the patient will have to use the product regularly throughout their lifetime.

## Conclusion

Immunologically active saliva substitute (IASS) significantly reduces subjective xerostomia scores measured using SXI and improves objective measurement of salivary flow using UWS among nasopharyngeal cancer survivors with xerostomia. Immunologically active saliva substitute (IASS) is significantly more effective in improving subjective and objective xerostomia measurements compared to non-immunologically active mouthwash. Additionally, this treatment is very safe, with superior side effect profiles.
